# Supplementary material for: Salicylic and Methyl Salicylic Acid Affect Quality and Phenolic Profile of Apple Fruits Three Weeks before the Harvest
Source: Plants (Basel). 2021 Aug 30;10(9):1807. doi: 10.3390/plants10091807 (PMC8467901; doi:10.3390/plants10091807)
Supplement: Supplementary file 1 [file plants-10-01807-s001.zip › plants-1336358-supplementary.pdf]

Table S1. Quantification of flavanols identified for the Spray#1 control and methyl salicylic acid (MeSA) and salicylic acid (SA) treatments for the apple fruit peel at the different sampling dates.

**Flavanols**	**Treatment**	**Content (g/kg dry weight)**				
		**Before spraying**	**After spraying**			
			**6 h**	**24 h**	**48 h**	**7 days**
Procyanidin dimers*a*	Control	1.87 ±0.02 a	2.32 ±0.10 a	2.03 ±0.08 a	2.06 ±0.21 a	1.87 ±0.12 a
	MeSA	1.84 ±0.01 a	2.39 ±0.23 a	2.58 ±0.25 b	2.24 ±0.21 a	1.96 ±0.07 a
	SA	1.83 ±0.01 a	2.18 ±0.12 a	2.33 ±0.03 ab	2.14 ±0.09 a	1.90 ±0.16 a
Procyanidin trimers*b*	Control	0.68 ±0.00 a	0.73 ±0.07 a	0.70 ±0.04 a	0.68 ±0.13 a	0.75 ±0.05 a
	MeSA	0.67 ±0.01 a	0.80 ±0.19 a	1.03 ±0.12 b	0.69 ±0.05 a	0.66 ±0.05 a
	SA	0.66 ±0.01 a	0.80 ±0.06 a	0.83 ±0.05 ab	0.65 ±0.04 a	0.59 ±0.10 a
Procyanidin tetramer	Control	0.11 ±0.00 a	0.16 ±0.01 a	0.29 ±0.06 a	0.17 ±0.03 a	0.16 ±0.03 a
	MeSA	0.11 ±0.00 a	0.19 ±0.10 a	0.20 ±0.03 a	0.11 ±0.02 a	0.14 ±0.02 a
	SA	0.11 ±0.01 a	0.19 ±0.03 a	0.14 ±0.02 a	0.11 ±0.02 a	0.12 ±0.04 a
Epicatechin	Control	0.50 ±0.01 a	0.65 ±0.02 a	0.72 ±0.06 a	0.61 ±0.04 a	0.56 ±0.03 a
	MeSA	0.51 ±0.00 a	0.66 ±0.07 a	0.65 ±0.02 a	0.63 ±0.05 a	0.57 ±0.02 a
	SA	0.51 ±0.01 a	0.63 ±0.04 a	0.58 ±0.03 a	0.64 ±0.03 a	0.59 ±0.04 a
Catechin	Control	0.02 ±0.00 a	0.02 ±0.00 a	0.03 ±0.00 a	0.02 ±0.00 a	0.03 ±0.00 a
	MeSA	0.02 ±0.00 a	0.02 ±0.00 a	0.02 ±0.00 a	0.02 ±0.00 a	0.03 ±0.00 a
	SA	0.02 ±0.00 a	0.03 ±0.00 a	0.02 ±0.00 a	0.02 ±0.00 a	0.02 ±0.00 a

Data are means ±standard error (n = 10)

Different letters indicate significant differences among the treatments within each flavanol and each sampling date ($p < 0.05$; Duncan tests)

[b]Sum of procyanidin dimer 1-5

[c]Sum of procyanidin trimer 1-3

Table S2. Quantification of flavonols identified for the Spray#1 control and methyl salicylic acid (MeSA) and salicylic acid (SA) treatments for the apple fruit peel at the different sampling dates.

Flavonols	Treatment	Content (g/kg dry weight)				
		Before spraying	After spraying			
			6 h	24 h	48 h	7 days
Quercetin-3-rutinoside	Control	12.91 ±1.91 a	9.88 ±1.06 a	16.82 ±3.57 a	12.01 ±0.86 a	18.97 ±2.83 a
	MeSA	9.57 ±1.91 a	13.07 ±1.19 a	13.60 ±2.15 a	11.78 ±3.99 a	13.05 ±0.68 a
	SA	9.69 ±1.70 a	15.81 ±2.68 a	19.11 ±1.35 a	12.37 ±3.58 a	13.10 ±1.15 a
Quercetin-3-galactoside	Control	234.6 ±16.4 a	198.6 ±26.6 a	298.2 ±51.5 a	223.1 ±19.4 a	199.8 ±12.9 a
	MeSA	174.9 ±19.5 a	262.2 ±34.4 a	259.1 ±35.5 a	193.4 ±42.8 a	325.6 ±18.0 b
	SA	179.3 ±29.2 a	276.7 ±44.2 a	345.1 ±23.3 a	202.9 ±40.1 a	216.6 ±10.6 a
Quercetin-3-glucoside	Control	11.35 ±0.84 a	10.07 ±0.85 a	15.03 ±2.28 a	10.93 ±0.64 a	11.04 ±0.72 a
	MeSA	8.64 ±1.09 a	12.46 ±1.09 a	12.70 ±1.57 a	9.85 ±1.78 a	14.68 ±1.43 b
	SA	9.27 ±1.45 a	13.49 ±1.62 a	16.38 ±1.07 a	10.81 ±2.11 a	10.86 ±0.50 a
Quercetin-3-xyloside	Control	58.90 ±2.92 a	10.07 ±0.85 a	78.03 ±11.27 a	60.61 ±3.01 a	53.64 ±3.89 a
	MeSA	43.95 ±2.86 a	12.46 ±1.09 a	72.27 ±7.91 a	52.90 ±6.06 a	68.75 ±0.92 b
	SA	50.33 ±6.23 a	13.49 ±1.62 a	88.20 ±6.14 a	55.07 ±5.64 a	54.15 ±0.69 a
Quercetin-3-arabinopyranoside	Control	10.38 ±0.70 a	8.90 ±0.93 a	11.57 ±1.76 a	9.16 ±0.61 a	8.89 ±0.62 a
	MeSA	7.38 ±0.80 a	6.98 ±3.02 a	10.74 ±1.40 a	8.14 ±1.60 a	12.84 ±0.71 b
	SA	8.12 ±1.48 a	12.33 ±1.41 a	13.38±0.89 a	8.43±1.57 a	9.60±0.40 a
Quercetin-3-arabinofuranoside	Control	58.41 ±2.49 a	66.96 ±3.77 a	86.11 ±13.13 a	68.56 ±4.66 a	60.20 ±4.89 a
	MeSA	47.59 ±2.69 a	72.00 ±7.65 a	77.89 ±8.57 a	59.03 ±7.24 a	74.09 ±1.15 b
	SA	52.63 ±7.19 a	73.85 ±7.35 a	98.89 ±6.71 a	60.69 ±5.60 a	59.71 ±1.34 a
Quercetin-3-rhamnoside	Control	816.0 ±63.7 a	876.3 ±36.4 a	1097.1 ±141.8 a	866.8 ±13.0 a	700.0 ±41.9 a
	MeSA	766.5 ±25.8 a	870.3 ±72.5 a	1017.4 ±113.2 a	748.8 ±54.5 a	882.5 ±38.5 b
	SA	757.2 ±76.7 a	937.9 ±54.6 a	1198.9 ±59.7 a	766.4 ±38.5 a	690.5 ±28.4 a

Data are means ±standard error (*n* = 10)

Different letters indicate significant differences among the treatments within each flavonol and each sampling date (p <0.05' Duncan tests)

Table S3. Quantification of hydroxycinnamic acids identified for the Spray#1 control and methyl salicylic acid (MeSA) and salicylic acid (SA) treatments for the apple fruit peel at the different sampling dates.

**Hydroxycinnamic acids**	**Treatment**	**Content (g/kg dry weight)**				
		**Before spraying**	**After spraying**			
			**6 h**	**24 h**	**48 h**	**7 days**
p-Coumaric acid hexosides[a]	Control	26.0 ±2.8 a	33.0 ±3.0 a	39.2 ±2.8 a	32.4 ±3.2 a	28.0 ±1.5 a
	MeSA	23.4 ±2.0 a	32.1 ±2.8 a	33.2 ±2.9 a	31.0 ±1.2 a	30.6 ±0.4 a
	SA	24.5 ±3.5 a	32.0 ±1.3 a	32.8 ±0.8 a	29.1 ±2.4 a	30.9 ±3.1 a
Chlorogenic acid	Control	119.6 ±6.4 a	134.2 ±1.7 a	107.2 ±10.6 a	120.3 ±9.2 a	113.0 ±3.4 a
	MeSA	118.1 ±2.4 a	119.0 ±6.0 a	148.0 ±4.7 b	117.0 ±8.2 a	145.3 ±7.6 b
	SA	117.2 ±2.6 a	118.1 ±4.7 a	115.2 ±5.5 a	115.1 ±2.6 a	108.5 ±3.4 a
Caffeoyl sinapoyl pentoside	Control	15.1 ±2.0 a	15.4 ±0.9 a	23.0 ±2.9 a	15.0 ±0.7 a	14.3 ±1.1 a
	MeSA	13.1 ±2.4 a	14.8 ±2.4 a	17.8 ±1.5 a	15.9 ±1.7 a	26.5 ±2.6 b
	SA	14.4 ±1.3 a	18.5 ±1.2 a	21.1 ±1.3 a	14.9 ±2.0 a	16.5 ±0.5 a
5-Caffeoylquinic acid	Control	13.9 ±2.4 a	12.4 ±2.9 a	24.0 ±4.1 b	13.5 ±3.7 a	13.9 ±1.8 a
	MeSA	9.4 ±0.6 a	17.3 ±6.3 a	18.2 ±1.6 ab	13.0 ±1.9 a	15.5 ±2.5 a
	SA	12.4 ±0.8 a	16.8 ±2.6 a	13.3 ±0.8 a	12.0 ±1.1 a	12.6 ±2.5 a
4-*p*-Coumaroylquinic acid	Control	0.1 ±0.0 a	0.1 ±0.0 a	0.1 ±0.0 b	0.1 ±0.0 a	0.1 ±0.0 a
	MeSA	0.0 ±0.0 a	0.1 ±0.0 a	0.1 ±0.0 ab	0.1 ±0.0 a	0.1 ±0.0 a
	SA	0.1 ±0.0 a	0.1 ±0.0 a	0.1 ±0.0 a	0.1 ±0.0 a	0.0 ±0.0 a

Data are means ±standard error (n =10)

Different letters indicate significant differences among the treatments within each hydroxycinnamic acid and each sampling date ($p < 0.05$; Duncan tests)

[a]Sum of *p*-coumaric acid hexoside 1 and 2
